# Evaluation of compliance to the World Health Organization’s five moments of hand hygiene: Cross-sectional observation of healthcare professionals

**DOI:** 10.4102/sajid.v36i1.255

**Published:** 2021-07-26

**Authors:** Thabiso L.A. Bale, Tendani S. Ramukumba, Lutendo S. Mudau

**Affiliations:** 1 Adelaide Tambo School of Nursing Science, Faculty of Science, Tshwane University of Technology, Pretoria, South Africa; 2 Department of Environmental Health, Faculty of Science, Tshwane University of Technology, Pretoria, South Africa

**Keywords:** hand hygiene compliance, five moments of hand hygiene, healthcare professionals, alcohol-based hand rub, hand washing, allied healthcare professionals, medical professionals, nurses

## Abstract

**Background:**

Human hands are home to thousands of microorganisms, which may be transmitted to surfaces that the hands come into contact with. When in contact with people who are ailing or have weakened immune systems, some of these microorganisms can cause infections and disease. Correct hand hygiene goes a long way in eradicating these potentially infective microorganisms and forms the cornerstone of infection prevention and control (IPC) within healthcare facilities and beyond. The healthcare industry is constantly challenged by healthcare-associated infections (HAIs) and their negative effects on patient safety and clinical outcomes. Hospitals in Pretoria are facing similar challenges posed by HAIs and there is no report available on compliance of healthcare professionals (HCPs) to the World Health Organization’s (WHO) ‘five moments of hand hygiene’. Healthcare professional’s compliance to all of the five moments of hand hygiene, particularly within the patient zone, is crucial in mitigating and reducing the spread of contact-based infections in the healthcare setting.

**Methods:**

A quantitative longitudinal design was used in a covert direct observation of HCP compliance to the WHO’s five moments of hand hygiene. The observations were conducted over 4 weeks in three hospitals, covering 25 wards, inclusive of four adult critical care units using the WHO’s ‘five moments of hand hygiene’ observation form.

**Results:**

A total of 1906 hand hygiene opportunities were directly observed in three hospitals. Hand hygiene compliance was 17.26% (*n* = 329). Allied health professionals had higher compliance (23.02%) than medical (19.26%) and nursing professionals (15.76%). The moment before patient contact had the lowest compliance (8.21%) as compared with all other moments.

**Conclusions:**

In general, HCPs had low compliance to the five moments of hand hygiene within the patient zone. Allied health professionals had higher compliance than medical and nursing professionals. Compliance in public hospitals was lower than in private hospitals. Critical care units had higher compliance compared to general wards. Healthcare professionals better complied to the moments meant for their safety as compared to those indicated for patient safety.

## Background

One of the risks for healthcare (hospitals in particular) in South Africa is the prevalence of healthcare-acquired infections (HAIs). Patient safety has become a leading priority for the healthcare industry in recent years^1^ towards patient-centred healthcare. An increase in HAIs threatens the achievement of this goal^2^ as HAIs are major contributors to patient morbidity and mortality globally.^3,4^ The most common mode of transmission of micro-organisms is through physical contact, which may be either direct or indirect between the host and person or surface carrying the micro-organism.^5^ In the absence of proper and adequate hand hygiene, hands of healthcare professionals (HCPs) play a significant role in the spread of HAIs. Contaminated hands can transfer pathogens from one touched surface to up to seven other objects or surfaces,^5,6^ thereby increasing the risk of infection transmission.

In Pretoria, which is part of the City of Tshwane metropolitan area in Gauteng, South Africa, HCPs work differently than in many other settings. For example, they attend to patients in more than one healthcare facility in a day. A great number of medical and allied practitioners perform remunerative work outside public service, moving between state, private and independent hospitals daily. Similarly, a large portion of nurses have a second job in a different healthcare facility, in addition to their regular employment (moonlighting) or work in multiple hospitals per week through nursing agencies. In such a setting, HCPs’ poor compliance to hand hygiene becomes a vehicle in spreading infections between facilities and increasing the incidence of HAIs.^6,7^

The World Health Organization (WHO) made hand hygiene in the healthcare setting a standard procedure globally.^7^ They further standardised moments when hand hygiene is indicated, which are: (1) before touching the patient, (2) before a clean or aseptic procedure, (3) after exposure to bodily fluids, (4) after touching the patient and (5) after touching the patient’s immediate surroundings.^8^ These moments are indicated in the care area called the patient zone. The patient zone is a clinical area where the HCP, the patient and the rendering of care meet.^9^ The area in the clinical facility that is outside the patient zone is called the healthcare zone and hand hygiene is not mandatory in this zone except for hygiene purposes.

It has been more than a decade since the publication of the WHO global hand hygiene guidelines for healthcare. There are global studies,^10,11,12,13,14,15^ describing the compliance to these guidelines, yet no study describing compliance to these guidelines has been published in South Africa. This study is aimed at describing HCPs’ compliance to the five moments of hand hygiene in Pretoria hospitals.

## Methods

There are 34 hospitals in Pretoria. Three of these hospitals were selected for the study based on the Hand Hygiene Self-Assessment Framework score (WHO), type of services offered, private or public service and willingness to participate in the study. A longitudinal quantitative survey was conducted over 4 weeks to observe the baseline compliance to the five moments of hand hygiene by HCPs in three hospitals in Pretoria. One of the three hospitals was a public hospital, while the other two were private hospitals. The three hospitals had a combined total of 465 licensed beds and 25 wards, inclusive of four adult critical care units (CCUs). The combined average occupancy of these facilities for the year 2018 was 70.76%. The difference between the public and private hospitals in this study was that the public hospital did not have a system to monitor the hand hygiene compliance of HCPs regularly, while the two private hospitals had an electronic system to collate all observations noted in each ward per professional category for each moment per month. The infection prevention and control (IPC) manager, who was a professional nurse, then provided the statistically analysed results to all stakeholders and departments every month.

The two private hospitals measured HCP hand hygiene compliance monthly. The combined total number of hand hygiene opportunities observed for the 12 months of 2018 was 15 015, with combined average compliance of 81.54%. These hospitals have at least one trained hand hygiene observer in each ward (who is part of the ward nursing employee) who captured the hand hygiene compliance in the ward either on paper or electronically.

For this study, each observation session was planned for 20 min with a 10-min extension or reduction based on the activities at the time of observation in each ward. The WHO’s observation form for hand hygiene compliance (Appendix 1)^9^ was used to record the observations. The study observers primarily observed one person at a time but were allowed to monitor two people at a time when possible. The observation sessions occurred during weekdays and during day shifts. Only the observations by the study observers were included in this research.

### Study population

The study population included all categories of nurses, all disciplines of medicine (as practised by each hospital) and all categories of allied HCPs (a person with special training, certification and licensing with responsibilities bearing on patient care), distributed across various shifts. No record of prior training in hand hygiene for these HCPs was obtained. The inclusion criteria were as follows: (1) HCPs who were registered with a professional accrediting body, (2) HCPs who were rendering healthcare service to a patient at the time of observation, (3) HCPs who were in the patient zone or with a patient and (4) the presence of one of the five moments of hand hygiene. Because of limited time and cost, a sample size of 1200 observations was calculated to achieve a confidence level of 95% with a non-response rate of 20%.

### Statistical analysis

The results were analysed using Statistical Package for the Social Sciences (SPSS) software. Descriptive statistics in the form of graphs and tables were used to summarise data into meaningful information. Inferential statistics were applied through the use of Pearson’s chi-square test to analyse the level of association between compliance and the following variables: facility, hospital type, ward, professional category, professional code and hand hygiene moments (Table 3).

In addition, a one-way analysis of variance (ANOVA) was conducted to assess the differences in observations received (Table 4). The results showed no significant difference in compliance (*p* > 0.05) based on the type of facility, facility type, professional category, professional code, hand hygiene moment or ward.

### Ethical considerations

Ethical approval was obtained from the Faculty of Science of Tshwane University of Technology in the north region of Gauteng province in South Africa (on 03 December 2018, reference number: FCRE 2018/09/007 (02) (SCI]), the Department of Health Tshwane District Office and the hospital administration of the hospitals in the study. This article followed all ethical standards for research without direct contact with human or animal subjects.

## Results

Three hospitals, which constituted 10% of the hospitals in Pretoria, were surveyed. The number of HCPs is undocumented as some work in more than one facility. Observations of hand hygiene compliance were conducted from 08 October to 15 November 2019 over a monitoring period of 40 days. Healthcare professionals knew that the observer was observing them for healthcare system delivery challenges but not that they were exclusively observed for their compliance to the five moments of hand hygiene. A total of 1906 direct observations related to hand hygiene opportunities were noted for three hospitals. Compliance was observed during 329 (17.26%) opportunities (Table 1). Compliance was higher in the CCUs than in the general wards for all HCP categories (Table 1).

**TABLE 1:** Observed compliance with the World Health Organization’s five moments of hand hygiene (overall study and comparison between critical care and general wards).

The WHO’s five moments of hand hygiene	All units			Critical care			General wards		
	Overall opportunities	Hand hygiene action taken		Overall opportunities	Hand hygiene action taken		Overall opportunities	Hand hygiene action taken	
		Compliant	%		Compliant	%		Compliant	%
**Before touching patient (moment 1)**	682	56	8.21	236	38	16.10	446	18	4.04
Allied	80	9	11.25	40	8	20.00	40	1	2.50
Medical	115	16	13.91	46	12	26.09	69	4	5.80
Nursing	487	31	6.37	150	18	12.00	337	13	3.86
**Before cleaning or aseptic procedure (moment 2)**	110	20	18.18	30	8	26.67	80	12	15.00
Allied	20	4	20.00	12	4	33.33	8	0	0.00
Medical	10	0	0.00	2	0	0.00	8	0	0.00
Nursing	80	16	20.00	16	4	25.00	64	12	18.75
**After exposure to bodily fluids (moment 3)**	115	39	33.91	43	18	41.86	72	21	29.17
Allied	14	4	28.57	10	3	30.00	4	1	25.00
Medical	12	5	41.67	4	3	75.00	8	2	25.00
Nursing	89	30	33.91	29	12	41.38	60	18	30.00
**After touching patient (moment 4)**	656	149	22.71	232	99	42.67	424	50	11.79
Allied	76	30	39.47	44	25	56.82	32	5	15.63
Medical	98	29	29.59	43	21	48.84	55	8	14.55
Nursing	482	90	18.67	145	53	36.55	337	37	10.98
**After touching patients’ immediate surrounding (moment 5)**	343	65	18.95	147	42	28.57	196	23	11.73
Allied	63	11	17.46	37	10	27.03	25	1	4.00
Medical	61	7	11.48	25	6	24.00	36	1	2.78
Nursing	219	47	18.95	85	26	30.59	135	21	15.56

**Grand total**	**1906**	**329**	**17.26**	**688**	**205**	**29.80**	**1218**	**124**	**10.18**

WHO, World Health Organization.

When hand hygiene compliance was present, an alcohol-based hand rub was used during 192 occasions (58.36%) and hands were washed with soap during 137 opportunities (41.64%).

Moment 1 (before touching the patient) had 682 observed opportunities, of which compliance was noted during 56 (8.21%) (Table 1). Overall compliance for moment 1 amongst HCPs including allied health professionals, medical and nursing staff was 11.25%, 13.91% and 6.37%, respectively (Table 1). For moment 2 (before a clean or aseptic procedure), the compliance per HCP category (Table 1) revealed that nurses and allied HCPs had a 20% compliance rate compared with medical practitioners (compliance rate of 0%).

Of the five moments of hand hygiene, HCPs had the second lowest compliance to moment 2.

Moment 3 (after exposure to bodily fluids) compliance (33.93%) was the highest amongst all the moments. Medical practitioners’ compliance was 41.69% followed by nursing personnel with 33.91% and Allied health professional with 28.57%. The compliance in general wards (29.17%) to this moment was the highest of all moments and second highest in the CCUs (41.86) (Table 1).

For moment 4 (after touching patient), the compliance was 22.71%. The results revealed that Allied health professionals had higher compliance of 39.47%, followed by medical and nursing with 29.95% and 18.67%, respectively. Moment 5 (after touching patient’s immediate surrounding) had an overall compliance of 18.95%. The results revealed that nurses had a higher compliance of 18.95%, followed by Allied health professionals with compliance of 17.46% and medical with compliance of 11.48% (Table 1).

The overall compliance to the five moments of hand hygiene was the lowest in the public facility compared to private facilities. At the public facility, HCPs complied during 6.88% of the 916 opportunities observed, whereas HCPs at the private facilities complied during 26.87% of the 990 opportunities observed. For the private hospitals, one hospital had 523 opportunities with a compliance rate of 26.76%, and the other had 467 opportunities with a compliance rate of 36.95%.

In examining the compliance per professional category, only 4 out of the 29 observed categories had compliance rates above 70%. Among these were cardiologists, with 73%, general surgeons, with 80% (Table 2). Further breakdown of compliance per professional code showed that allied health professionals had the highest compliance rate amongst the three groups. The allied health personnel had a compliance rate of 28% to the five moments of hand hygiene, followed by medical personnel with 19% and nursing personnel with 16%.

**TABLE 2:** Comparison of compliance to the five moments of hand hygiene in private versus public setting: Breakdown per professional code (*p* = 0.010).

Professional category	Private			Public			Overall		
	Opportunities	Hand hygiene done	Compliance (%)	Opportunities	Hand hygiene done	Compliance (%)	Opportunities	Hand hygiene done	Compliance (%)
Any other health professional (undescribed)	7	0	0.00	39	1	2.56	46	1	2.17
Clinical technologist (cardiology)	7	0	0.00	-	-	-	7	0	0.00
Clinical Technologist (neurology)	2	0	0.00	-	-	-	2	0	0.00
Dialysis staff (clinical technologist or nurse)	53	18	33.96	-	-	-	53	18	33.96
Dietician	9	1	11.11	-	-	-	9	1	11.11
Environmental health (community service)	-	-	-	2	0	0.00	2	0	0.00
Laboratory technician (phlebotomists and nurses)	41	12	29.27	-	-	-	41	12	29.27
Physiotherapist	55	15	27.27	16	0	0.00	71	15	21.13
Psychologist	1	0	0.00	-	-	-	1	0	0.00
Radiographer	20	11	55.00	-	-	-	20	11	55.00
Social worker	1	0	0.00	-	-	-	1	0	0.00
Bachelor of clinical medical practice (student)	-	-	-	31	2	6.45	31	2	6.45
Cardiologist	11	8	72.73	-	-	-	11	8	72.73
Cardiothoracic surgeon	20	6	30.00	-	-	-	20	6	30.00
DR (discipline unknown)	18	2	11.11	110	6	5.45	128	8	6.25
General surgeon	15	12	80.00	-	-	-	15	12	80.00
Medical student	-	-	-	4	0	0.00	4	0	0.00
Nephrologist	24	6	25.00	-	-	-	24	6	25.00
Neurologist	1	1	100.00	-	-	-	1	1	100.00
Orthopaedic surgeon	2	0	0.00	-	-	-	2	0	0.00
Paediatrician	1	1	100.00	-	-	-	1	1	100.00
Physician	33	10	30.30	-	-	-	33	10	30.30
Pulmonologist	16	0	0.00	-	-	-	16	0	0.00
Radiologist	2	0	0.00	-	-	-	2	0	0.00
Vascular surgeon	8	3	37.50	-	-	-	8	3	37.50
Enrolled nurse (EN)	148	33	22.30	12	3	25.00	160	36	22.50
Enrolled nursing auxiliary (ENA)	159	30	18.87	293	22	7.51	452	52	11.50
Registered nurse (RN)	325	96	29.54	309	20	6.47	634	116	18.30
Student nurse	11	1	9.09	100	9	9.00	111	10	9.01

**Grand total**	**990**	**266**	**26.87**	**916**	**63**	**6.88**	**1906**	**329**	**17.26**

## Discussion

To our knowledge, this study was the first in South Africa to describe HCPs’ compliance to the WHO’s five moments of hand hygiene in public and private hospitals. However, several international studies have reported on this.^10,11,12,13,14,15^ The results of this study showed poor compliance to hand hygiene by HCPs, similar to the results from other studies worldwide.^13,14,15^ In facilities where hand hygiene compliance was observed consistently before our study, it was performed by observers from within the specific ward leaving room for the Hawthorne effect^16,17^ on reported compliance. The results showed a compliance rate of less than 50% of the self-reported compliance at the two private hospitals. This may have been because of the hand hygiene compliance rate being amongst the key performance indicators (KPIs) linked to management incentive bonuses.

This link to incentive may have led to the selective observation that focused more on compliant moments to maintain the target for incentive, and hence the variance between the self-reported compliance and our results.

The statistical analysis (Table 3) revealed that HCPs’ level of compliance to hand hygiene had a significant association with facility and facility types, professional code and category, hand hygiene moment and wards where observations were conducted. This corroborated with Novoa et al.’s finding that HCPs had higher compliance rates in the CCU compared to surgical wards.^15^ The one-way ANOVA analysis results showed no variance in parameters, implying that the same interventions would be needed to address non-compliance in all settings of this study.

**TABLE 3:** Level of overall compliance to the five moments of hand hygiene by healthcare professionals.

Variable	Number	%	Proportion complied		*p*
			Number (*n*)	%	
Total observations	1906	-	329	17.26	-
**Professional code**	-	-	-	-	0.012
Allied healthcare	252	13.22	58	23.02	-
Medical	296	15.53	57	19.26	-
Nursing	1358	71.25	214	15.76	-
**Wards**	-	-	-	-	2.34
Critical care	699	36.67	209	29.90	-
General wards	1207	63.33	120	9.94	-
**Facility type**	-	-	-	-	8.45
Private	990	51.94	266	26.87	-
Public	916	48.06	63	6.88	-
**Moments**	-	-	-	-	1.32
Before touching patient	682	35.78	56	8.21	-
Before clean or aseptic procedure	110	5.77	20	18.18	-
After exposure to bodily fluids	115	6.03	39	33.71	-
After touching patient	656	34.42	149	22.71	-
After touching patient immediate surrounding	343	18.00	65	18.95	-

Hand hygiene compliance was almost 30% higher in the CCUs than in the general wards, including the emergency centre (Table 1). These results were similar to those of Novoa et al. who reported a higher hand hygiene compliance rate in the CCU compared to surgical wards.^15^ This could be because of the nature of the allocation of staff and activities, as well as HCPs’ perception of CCUs. We noted with concern that the two moments during which HCPs must protect the patient, that is, before patient contact and before cleaning or aseptic procedures, their hand hygiene compliance was the lowest compared to those moments after being with the patient^18^ and this was noted for private and public hospital settings (Table 3) in our study. Healthcare professionals in the public hospital setting had lower compliance to the five moments of hand hygiene compared to the private hospital setting.

In the private hospitals, continued observation of hand hygiene practices was part of their performance indicators and the observation system had regular ward observers. They reported a higher compliance rate compared to the study results. Additional hand hygiene opportunities were noted which were not included in the WHO’s five moments such as handwashing after the random ringing of a bell that summoned everyone to stop what they were doing and line up to wash their hands with soap and water at the basins near them.

The public hospital had no concurrent observation while we collected our data. A point of concern regarding the low compliance was the rotation of a considerable number of students and interns from various disciplines and various universities and colleges who were not compliant with the five moments of hand hygiene with their mentors (clinical staff in the hospitals). Clinical mentors are setting a bad example based on their poor compliance to the five moments of hand hygiene in the presence of students and subordinates.

Changes in behaviour, which are a result of the reinforcement learning process involving the targeting of evolved motives and changes to behaviour setting, are produced by a set of behaviour control mechanisms (automatic and motivated).^19^ The repeated automatic non-compliance to the five moments of hand hygiene acts as a reinforcement of behaviour that is dangerous to patients. In New Zealand, results from the first national hand hygiene compliance audit for 2012 concurred with this, stating that, ‘just as positive role-modeling can have a very positive impact on the hand hygiene practice of others, negative role-modeling can undo a lot of hard work’.^20^ If the compliance to the five moments of hand hygiene as a behaviour of healthcare practice is not taught in these future HCPs while they are in the formation phase, changing this behaviour would become a healthcare challenge at a later stage in their profession as reported in another study^21^ that students in health science copy what their seniors do. This is a noteworthy point for teaching hospitals to remember in imparting and fostering conducive behaviours for patient safety in current and future HCPs.

The results shed light on the hand hygiene compliance of HCPs per category or discipline. For moments 2 and 5, nurses had better compliance compared to those in medical disciplines which was similar to another study.^22^ Except for moments 2 and 5, the overall results showed that medical professionals had a higher compliance to hand hygiene than nurses. These results were similar to other studies^23,24,25^ where nurses had lower compliance to hand hygiene than doctors. Interestingly, the allied HCPs’ compliance was higher compared to both the nurses and medical personnel. We could not find a study that reported allied HCPs’ compliance to hand hygiene, but Staines et al. reported that physiotherapists demonstrated better hand hygiene compliance compared to both nurses and medical doctors.^24^

### Before patient contact

Hand hygiene compliance is crucial for the moment before patient contact to prevent the transmission of pathogens from the HCP to the patient, and ultimately to protect the patient against colonisation and exogenous infection carried on HCPs’ hands.^9^

The moment before touching the patient had the lowest compliance of all the moments as was reported in other studies.^23,26,27,28^ The compliance to this moment in the general ward was lower than in the critical care. We observed that the HCPs often failed to perform hand hygiene even when an alcohol-based hand rub was readily available on the care trolley in front of them.

Medical staff showed a higher compliance to this moment, as nurses would remind them or offer the alcohol-based hand rub agent to them. However, nurses would not perform hand hygiene and would continue to touch the patient. This poses a risk when the HCP touching the patient has been to other wards or facilities (allied health workers and medical staff) and may be carrying pathogens from the last touched surface, patient or object in one ward or facility to a patient in another ward or facility.

In most cases, a nurse taking care of four patients in a single room would move with the same equipment from one patient to the next without performing hand hygiene, and without disinfecting the blood pressure cuff or pulse oximetry probe with the surface disinfectant spray available on the trolley. The same was observed with physician rounds in wards – physicians would touch patients without performing hand hygiene after touching patient files and other objects. The laboratory staff usually complied with this moment and set up a clean or sterile field for the collection of blood specimens. Even though nurses had four to five times more indications for this moment, they had the lowest hand hygiene compliance to the moment before touching the patient.

### Before clean or aseptic procedures

The compliance to this moment was poor, similar to another study.^23^ Both nurses and allied HCPs showed better compliance than medical doctors. Lau et al. in a study that compared compliance to five moments of hand hygiene between medical and nursing students found that nursing students had better compliance than medical students.^22^ It is concerning that those in the medical profession had zero compliance to this moment when they were not in the theatre environment. Medical professionals had 80% of the indications for this moment in the general ward and the remaining 20% in the CCU. In the general ward, this moment was usually related to the insertion of intravenous infusion (IV) lines. The lack of hand hygiene before inserting an IV line and compliance to an aseptic procedure is one of the contributors to bloodstream infections that are HAIs.^5^ The results for moment 2 were similar to Novoa et al.’s finding that, in situations where the risk for infection was high, hand hygiene compliance was lower compared to situations where the risk was intermediate.^15^

Four procedures that have a direct impact on HAIs by contributing to central line-associated bloodstream infections (CLABSI) featured prominently. The first of these was accessing the dialysis catheter that is placed in the large blood vessel. Performing this procedure without performing hand hygiene may lead to contamination of the dialysis catheter and cause CLABSI as the micro-organisms would travel down the catheter into the bloodstream through the insertion site.^5^ The risk of hospitalisation for infections related to the dialysis catheter, and death, is two to three times higher for those without a dialysis catheter or graft.^29^

The second procedure was administering IV medication, especially through the central venous pressure line. We also observed how the ports were often not cleaned with an antiseptic swab before administering IV medication. We saw that HCPs in this study were only 25% compliant to hand hygiene before administering IV medication. This indicated that there was a tendency to administer medication to patients without adhering to safety protocols of hand hygiene before administering IV medications.

The third procedure was obtaining blood specimens. The laboratory personnel (nurse or phlebotomist) usually performed hand hygiene upon arrival at the bed space of the patient.

This would be followed by preparing the working space and touching the patient to locate the site for venipuncture. Hand hygiene is indicated again immediately before donning sterile gloves to perform venipuncture. At this point, hand hygiene was missed by this category of HCP. Failing to perform hand hygiene during this moment leads to contamination and has an impact on the culture results. When specimens for blood culture are drawn from the central venous pressure line and hand hygiene is omitted, it leads to contamination of the port and the sample taken.

The fourth procedure was the administration or changing of the total parenteral nutrition (TPN) bag and line. Total parenteral nutrition is high in lipids and carries a great infection risk^30,31^ if the procedure is not conducted aseptically.

Studies^30,31,32^ have shown that the incidence of bloodstream infections is high in patients on TPN, which warrants that each instance of commencing, changing or terminating the TPN be performed with strict adherence to the aseptic principles. In general, the management of intravenous lines was often associated with the absence of hand hygiene. This is concerning as intravenous lines, inclusive of central lines, contribute significantly to HAIs. Hospitals were provided with feedback on these findings soon after the study was concluded, to enable them to implement corrective training interventions.

### After exposure to bodily fluids

In most cases, the moment of hand hygiene after exposure to bodily fluids followed the moment before the clean or aseptic procedure. We observed higher compliance to this moment in both CCUs and general wards compared to the moment before clean or aseptic procedures, in line with practices reported in other studies.^27,28^ This showed that HCPs were more likely to protect themselves than to protect patients. In a study to explore reasons for poor hand hygiene amongst hospital workers, HCPs reported that hand hygiene was mostly performed after tasks perceived to be dirty.^33^ The authors further reported that personal protection (in the form of performing hand hygiene) appeared to be more important than patient safety.^33^ We observed that allied healthcare workers had lower compliance than nurses and doctors. Doctors had the highest compliance to this moment amongst all the HCPs, while they had no compliance to the moment before clean or aseptic procedures. This may have been because of the previously stated belief about protecting oneself from what is perceived to be dirty or contaminating

### After touching patients

This moment was observed to have the second highest overall compliance amongst the five moments. In the CCUs, this moment had the overall highest compliance with allied HCPs leading the compliance rates. Numerous studies^23,24^ have reported overall higher compliance to hand hygiene by physiotherapists compared to doctors and nurses. The compliance in the general wards, however, was lower because of the HCPs’ routine activities in the wards amongst four to six patients in the same room providing little opportunity for HCPs to perform hand hygiene. Even though alcohol-based hand rub was available at each patient zone in a few wards, HCPs did not use it and continued to move to the next activity (e.g. writing in the patient file or moving to touch the next patient).

### After touching patients’ immediate surroundings

The moment after touching the patient’s immediate surroundings had a low compliance rate, similar to moment 2. Low compliance to this moment poses a risk in the case of medical professionals and allied HCPs who go to another ward or hospital when they leave the patient’s bedside, carrying pathogens from the last patient or the patient’s immediate surroundings on their hands. The hands of HCPs have been reported to be vectors in the transmission of fomites (when hand hygiene is absent or inadequate) from one source to about seven other touched objects or people.^6^

## Conclusion

It can be concluded that HCPs have low compliance to the five moments when hand hygiene is indicated by WHO. The moment before a clean or aseptic procedure carries considerable risk, especially with low hand hygiene compliance. Healthcare professionals had higher compliance rates after exposure to bodily fluids. Hand hygiene compliance was higher in the CCUs compared to the general wards. Compliance in the public hospital was lower than in the private hospitals. The observation of hand hygiene compliance by hospital-appointed observers did not correspond with findings of the study, and future research is needed to determine whether observations by hospital-appointed observers are helpful. The movement of HCPs between multiple facilities within the same day carries a high risk of spreading pathogens carried through the hands of HCPs who have displayed poor hand hygiene compliance. Healthcare professionals experience barriers to compliance to hand hygiene that were not explored in this study. Further research is needed to describe HCPs’ barriers to compliance to the five moments of hand hygiene.
